# Controlled Synthesis of Platinum and Silver Nanoparticles Using Multivalent Ligands

**DOI:** 10.3390/nano12132294

**Published:** 2022-07-04

**Authors:** Suguna Perumal, Raji Atchudan, Eckart Rühl, Christina Graf

**Affiliations:** 1 Physikalische Chemie, Institut für Chemie und Biochemie, Freie Universität Berlin, 14195 Berlin, Germany; suguna.perumal@gmail.com; 2 Department of Chemistry, Sejong University, Seoul 143747, Korea; 3 School of Chemical Engineering, Yeungnam University, Gyeongsan 38541, Korea; atchudanr@yu.ac.kr; 4 Department of Chemistry and Biotechnology, Darmstadt University of Applied Sciences, 64295 Darmstadt, Germany

**Keywords:** Pt(acac)_2_, Ag(acac)_2_, monodisperse nanoparticles, size distribution, digestive ripening, multivalent ligands

## Abstract

Here, the controlled formation of platinum nanoparticles (PtNPs) and silver nanoparticles (AgNPs) using amine-functionalized multivalent ligands are reported. The effects of reaction temperature and ligand multivalency on the growth kinetics, size, and shape of PtNPs and AgNPs were systematically studied by performing a stepwise and a one-step process. PtNPs and AgNPs were prepared in the presence of amine ligands using platinum (II) acetylacetonate and silver (I) acetylacetonate, respectively. The effects of ligands and temperature on the formation of PtNPs were studied using a transmission electron microscope (TEM). For the characterization of AgNPs, additionally, ultraviolet-visible (UV-Vis) absorption was employed. The TEM measurements revealed that PtNPs prepared at different temperatures (160–200 °C, in a stepwise process) are monodispersed and of spherical shape regardless of the ligand multivalency or reaction temperature. In the preparation of PtNPs by the one-step process, ligands affect the shape of the PtNPs, which can be explained by the affinity of the ligands. The TEM and UV-Vis absorption studies on the formation of AgNPs with mono-, di-, and trivalent ligands showed narrower size distributions, while increasing the temperature from 80 °C to 120 °C and with a trivalent ligand in a one-step process.

## 1. Introduction

In the emerging and attractive world of nanomaterials, nanosized particles of noble metals (platinum, silver, and gold) have attracted much attention in recent years due to their applications in various fields, such as catalysis [1,2,3,4], optics [5,6,7], biological applications [8,9,10,11,12,13], and biosensors [14,15,16,17,18]. Platinum nanoparticles (PtNPs) have shown unique and promising electrocatalytic properties in fuel cells [19,20,21,22]; nonlinear optical properties [23,24] in electronic devices [25,26]; as electrochemical biosensors for the detection of aquatic products [27], glucose [28], and glutamate in food products [29], and for the detection of reactive oxygen species [30,31]. Like PtNPs, silver nanoparticles (AgNPs) have also received much attention in the fields of catalysis in organic synthesis [32,33,34], optics [35,36], and antibacterial agents [37,38,39,40], and in biomedical devices [41,42,43].

In general, PtNPs and AgNPs are synthesized using single-phase [44,45,46,47] and two-phase transfer methods [44,48,49]. By carefully adjusting the reaction parameters (such as reaction time, temperature, reagents, and capping agents), the size, shape, and quality of the PtNPs and AgNPs can be controlled [44,45,46,47,48]. Much work has been done to develop a synthetic route to obtain monodispersed and shape-controlled nanoparticles [44,45,46,47,48]. Mostly, organometallic precursors have been used for the preparation of bimetallic nano-alloy particles [50,51,52]. To the best of our knowledge, only a few reports are available on the preparation of PtNPs and AgNPs using platinum (II) acetylacetonate (Pt(acac)_2_) [53,54,55] and silver (I) acetylacetonate (Ag(acac)) [44,56] as precursors, respectively. 

The capping agent plays an essential role in the formation of nanoparticles; mostly, thiols [57,58,59], amines [60,61,62], and polymers [63,64,65] are used as capping agents. Recently, multivalent ligands have been the focus of research because of their potential uses in biological applications [65,66]. Many biological systems, such as proteins, have amine groups in their structure, and hence, can stabilize PtNPs and AgNPs. The use of multivalent ligands is expected to enhance the efficiency as compared with the monovalent ligands [65,66]. Due to multivalent ligands’ high avidity and specificity, they can act as potent inhibitors for pathogen and SARS-CoV-2 infection [67,68]. The multivalent ligand system binding on nanoparticles enhances the stability of the nanoparticles [67,69]. We published a study on the kinetics of the exchange of dye molecules bound to the surface of gold nanoparticles by thiol functionalized multivalent ligands [70]. The binding modes of thiol and multivalent ligands on different sizes of gold nanoparticles have been systematically investigated [70]. The reaction rates increased with an increase in the size of the gold nanoparticles, and they were faster for multivalent ligands as compared with monovalent ligands.

Here, the morphologies of PtNPs and AgNPs were controlled by amine-functionalized mono- and multivalent ligands. For the synthesis of multivalently stabilized PtNPs and AgNPs, Pt(acac)_2_ and Ag(acac) were used, respectively. In the stepwise process, the PtNPs and AgNPs were synthesized between 80 °C and 200 °C by increasing the reaction temperature steadily. Through the one-step process, PtNPs and AgNPs were prepared at 200 °C and 120 °C, respectively. The prepared amine-stabilized PtNPs and AgNPs were systematically investigated using transmission electron microscopy (TEM) and ultraviolet-visible (UV-Vis) absorption spectroscopy. 

## 2. Experimental Section

### 2.1. Materials and Methods

Pt(acac)_2_ (97%), Ag(acac) (97%), octyl ether (OE) (99%), dodecylamine, 1,2-hexadecandiol (HD) (90%), and all dry solvents used for preparation were purchased from Aldrich Chemical Co, Darmstadt, Germany. and used as received. Ethanol p.a. and dichloromethane (CH_2_Cl_2_) were purchased from VWR, Darmstadt, Germany and used as received. Column chromatography was performed using 40–63 µm mesh silica gel. Thin-layer chromatography (TLC) was performed using TLC silica gel 60f_254_ plates which were purchased from Merck, Darmstadt, Germany. ^1^H and ^13^C nuclear magnetic resonance (NMR) spectra were recorded using a Bruker DMX-NMR spectrometer, Rheinstetten, Germany, operating at 400 and 100 MHZ for ^1^H and ^13^C, respectively. The spectra were measured in chloroform-d purchased from Deutero GmbH, Kastellaun, Germany; chemical shifts were referenced relative to the deuterated solvent’s residual proton or carbon signal as δ = 7.26 for the ^1^H and δ = 77.00 for the ^13^C spectra. Before synthesis, the glassware was washed with aqua regia (3:1, conc. HCl/conc. HNO_3_) and distilled water. Caution is advised since, the aqua regia should be handled with extreme care.

### 2.2. Synthesis of Multivalent Ligands

**Figure 1:**
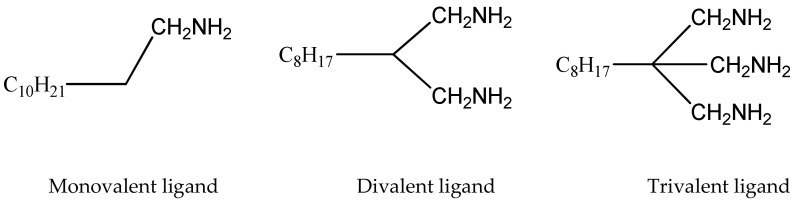
Chemical structures of dodecylamine (monovalent ligand), 2-octylpropane-1,3-diamine (divalent ligand), and 2-(aminomethyl)-2-octylpropane-1,3-diamine (trivalent ligand).

The structures of the monovalent ligand (dodecylamine) and flexible di- and trivalent amine ligands were prepared as described in [71] and are shown in Figure 1. As the di- and trivalent amine ligands were prepared using reported work, the synthesis procedure is not shown here. However, the amine ligands were characterized using NMR and the chemical shifts of divalent amine and trivalent ligands from H-NMR and C-NMR are shown in the Supplementary Materials. 

### 2.3. Synthesis of Amine-Coated PtNPs and AgNPs at Different Temperatures by Performing a Stepwise Process

**Scheme 1:**
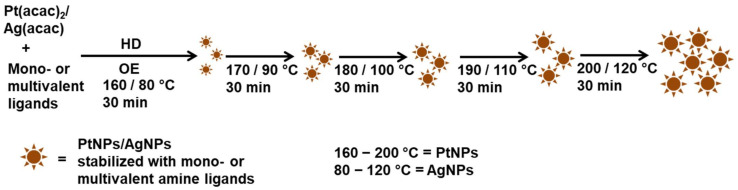
Schematic representation of the synthesis of AgNPs and PtNPs with mono- and multivalent ligands using a stepwise process.

All nanoparticles were synthesized under an argon atmosphere. The PtNPs stabilized with mono- and multivalent amine ligands were prepared as shown in Scheme 1. Typically, to 0.02 mmol of Pt(acac)_2_ or Ag(acac) in 1 mL of OE, 0.04 mmol of mono- or multivalent amine ligands and 0.20 mmol of HD were added. The solutions were heated to 80 °C and 160 °C for 30 min with an increasing temperature rate of 0.3 K/min for AgNPs and PtNPs, respectively. After 30 min, the temperature was raised by 10 K/30 min until the temperature reached 120 °C and 200 °C for AgNPs and PtNPs, respectively. The reaction mixtures were maintained at 120 °C (AgNPs) and 200 °C (PtNPs) for 30 min, respectively. Samples were collected at 170 °C, 180 °C, 190 °C, and 200 °C and at 90 °C, 100 °C, 110 °C, and 120 °C for PtNPs and AgNPs, respectively. The collected nanoparticles were purified by the addition of ethanol and used for characterization. 

### 2.4. Synthesis of Amine-Coated PtNPs by Performing a One-Step Process

**Scheme 2:**
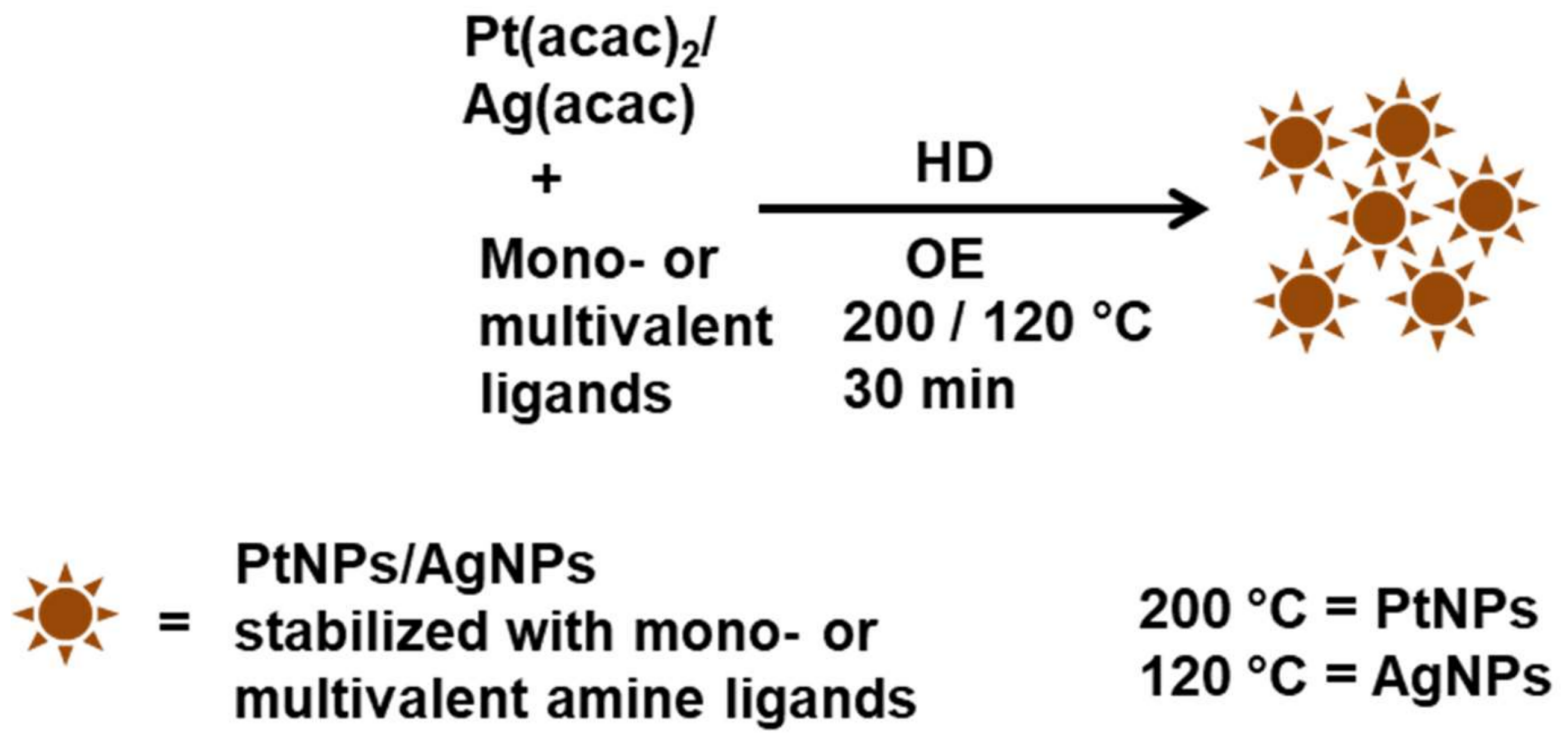
Schematic representation of the synthesis of AgNPs and PtNPs with mono- and multivalent ligands using the one-step process.

During the synthesis of nanoparticles, the argon atmosphere was maintained. The mono- and multivalent amine ligand-stabilized PtNPs were prepared in the same way unless specifically mentioned (Scheme 2). To Pt(acac)_2_ (0.02 mmol) in OE (1 mL), mono-, di- or trivalent amine ligands (0.04 mmol) were added, and the reaction mixture was heated to 200 °C. Then, HD (0.20 mmol) in 1 mL of OE was quickly injected into this mixture. The color of the dispersion turned to blackish brown immediately for monovalent amine and within 5 min in the case of di- and trivalent amine ligands, indicating the spontaneous formation of PtNPs. Then, the reaction mixture was maintained at 200 °C for 30 min for tri- and divalent ligands and 5 min for monovalent amine ligands. After the specified time had elapsed, the reaction mixture was cooled naturally to room temperature and the nanoparticles were precipitated by the addition of ethanol. Amine-stabilized PtNPs were isolated using centrifugation at 1000× *g* for 10 min. PtNPs stabilized with amine ligands were washed three times with ethanol. 

To prepare the amine-stabilized AgNPs, in the procedure of PtNPs synthesis, Pt(acac)_2_ was replaced by Ag(acac), and the reaction mixture was heated to 120 °C instead of 200 °C. 

The AgNPs and PtNPs stabilized with amine ligands were named as follows: M-AgNPs are AgNPs stabilized by monovalent amine ligands, D-AgNPs are AgNPs stabilized by divalent amine ligands, and T-AgNPs are AgNPs stabilized by trivalent amine ligands. Analogously, PtNPs were named: M-PtNPs, D-PtNP, and T-PtNPs. These sample designations are used in the following sections.

### 2.5. Characterization of the Samples

Samples for characterization by TEM were prepared by dipping carbon-coated copper 400-mesh grids (Quantifoil, Großlöbichau, Germany) in M-AgNPs, D-AgNPs, T-AgNPs, M-PtNPs, D-PtNPs, and T-PtNPs dispersions in CH_2_Cl_2_. High magnification TEM images were recorded by using a Philips CM 200 FEG microscope and Technai F20 FEI microscope (FEI, Dreieich, Germany) at 200 kV. Around 800 nanoparticles were analyzed from each sample to determine the average diameter and the polydispersity of the nanoparticles using the software Simple PCI (C-Images).

For UV-Vis absorption spectroscopy, absorption spectra of M-AgNPs, D-AgNPs, and T-AgNPs were dispersed in CH_2_Cl_2_ and measured using 1.00 cm SUPRASIL quartz cells (Hellma, Müllheim, Germany) in a Lambda 19 spectrophotometer (PerkinElmer LAS, Rodgau, Germany).

## 3. Results and Discussion

### 3.1. Temperature and Ligand Effects on the Formation of PtNPs and AgNPs by Performing the Stepwise Process

To study temperature and ligand effects on the formation of PtNPs, Pt(acac)_2_ in OE was heated for about 30 min with mono- or multivalent ligands and HD at different temperatures (160–200 °C). Subsequently, aliquots from the reaction were collected at each temperature (160 °C, 170 °C, 180 °C, 190 °C, and 200 °C). The reaction temperature was fixed between 160 °C and 200 °C because the formation of PtNPs was observed from 160 °C and decomposition of PtNPs was observed when the reaction mixture was heated above 200 °C. Further, the reaction time was fixed to 30 min because heating of the reaction mixture for longer times resulted in aggregation.

**Figure 2:**
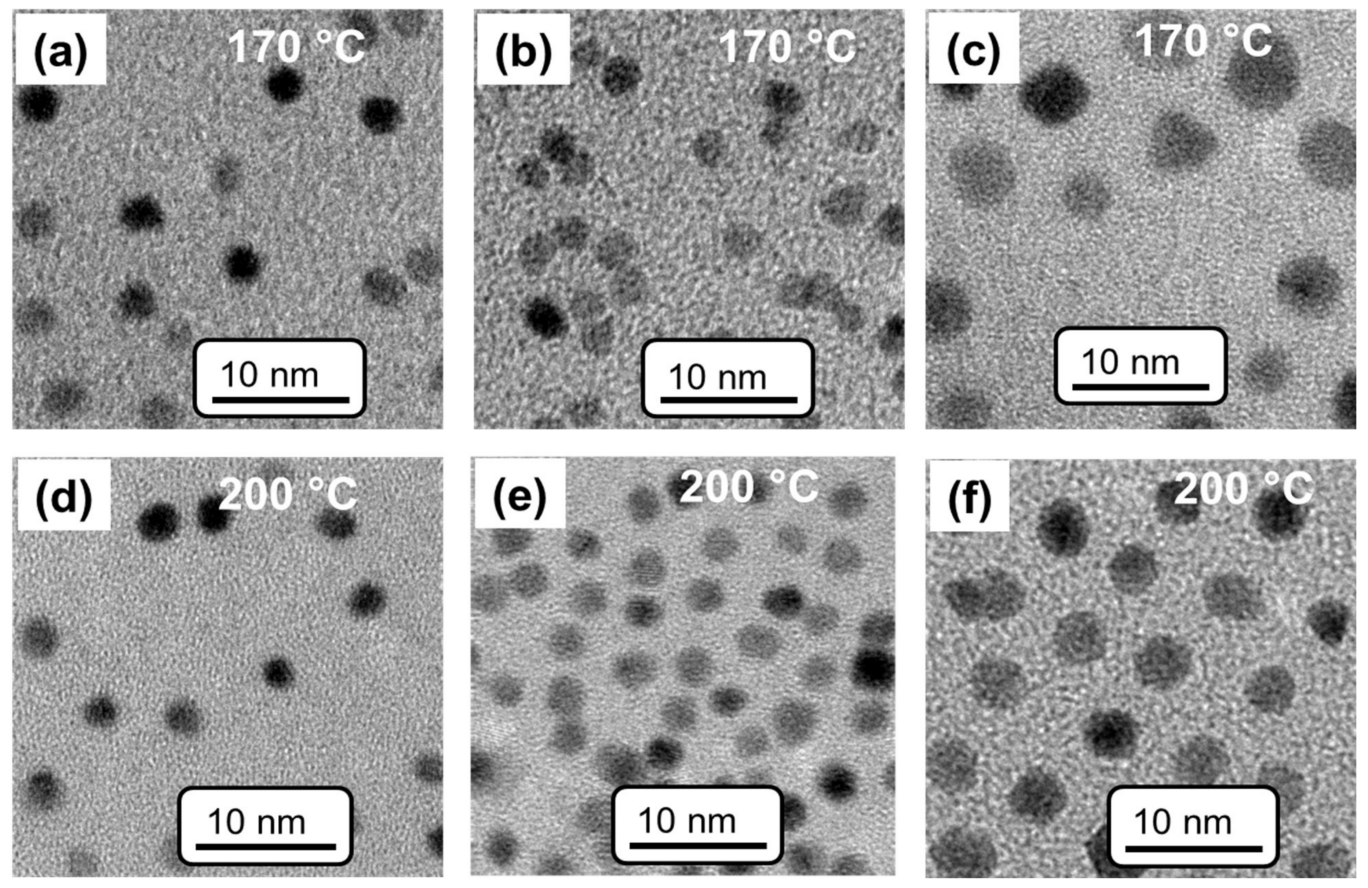
High magnification TEM images of M-PtNPs, D-PtNPs, and T-PtNPs: (**a**–**c**) at 170 °C; (**d**–**f**) and at 200 °C.

The TEM images of M-PtNPs, D-PtNPs, and T-PtNPs at 170 °C and at 200 °C are displayed in Figure 2a–c and Figure 2d–f, respectively. At the initial stage of the reaction, the mixture was colorless, then, it turned to a yellow color at 160 °C, and then into a light brown color at 170 °C, which indicated the formation of PtNPs. At 180 °C, the reaction mixture turned to a dark brown color. During the synthesis of M-PtNPs, D-PtNPs, and T-PtNPs, it was observed that the precursors completely dissolved at 170 °C. The TEM images of M-PtNPs, D-PtNPs, and T-PtNPs prepared at 170 °C, and 200 °C are shown in Figure 2. The particle sizes of M-PtNPs, D-PtNPs, and T-PtNPs prepared at 170 °C were determined to be 2.5 ± 0.6, 2.2 ± 0.8, and 3.0 ± 0.4 nm, respectively. M-PtNPs, D-PtNPs, and T-PtNPs prepared at 200 °C showed slightly larger particle sizes of 2.8 ± 0.5, 2.3 ± 0.4, and 3.7 ± 0.5 nm, respectively.

The polydispersity index of PtNPs prepared at 200 °C was determined to be about ±0.5, which was narrower than for those particles prepared at 170 °C (±0.6 to ±1.4). This indicated that reaction temperature controlled the growth of the particles. This finding is further supported by the observation of change in reaction mixture color during the synthesis of PtNPs. The reaction mixture was light brown only at 170 °C, suggesting the formation of PtNPs started at this temperature. The size differences among the M-PtNPs, D-PtNPs, and T-PtNPs prepared at 170 °C are found within the error range, indicating that the ligands do not significantly affect the size of PtNPs at this temperature. However, the size of the T-PtNPs from Figure 2c is slightly larger than that of the M-PtNPs and D-PtNPs. In addition, a larger size of T-PtNPs was observed at 200 °C than for M-PtNPs and D-PtNPs. This can be explained by considering the binding of sterically hindered trivalent ligands on the surface of PtNPs. Evidently, trivalent ligands cannot be bound with all three amine groups to the curved surface of PtNPs. This was in agreement with previous work on trivalent thiol ligands bound to gold nanoparticles, where a similar situation has been reported [70]. This was due to the steric hindrance of trivalent ligands, where only two thiol groups were bound to the gold surface, and as a result, trivalent ligands showed similar ligand exchange rates as compared with divalent thiol ligands on gold nanoparticles of the same size [70]. 

Studies on temperature and ligand effects on the formation of M-PtNPs, D-PtNPs, and T-PtNPs using different batches and following the same procedure were highly reproducible. The syntheses yielded almost identical particle sizes within ±0.05 nm. 

Subsequently, the temperature effects on the formation of M-AgNPs, D-AgNPs, and T-AgNPs were systematically studied. AgNPs could be characterized by performing UV-Vis absorption spectroscopy because AgNPs exhibit distinct surface plasmon bands around 400 nm [72,73]. Here, the temperature and ligand effects of M-AgNPs, D-AgNPs, and T-AgNPs were investigated using the full width at half maximum (FWHM) from the UV-Vis absorption spectrum, the spectral maxima, and the size distribution derived from TEM measurements. The reaction temperature was fixed between 80 °C and 120 °C. This temperature range is because particle formation is observed at 80 °C. Further, when the reaction mixture was heated above 120 °C, decomposition of AgNPs was observed. Further, the reaction time was also limited to 30 min, as in the case of PtNPs, because heating of the reaction mixture to temperatures above 120 °C resulted in aggregation of AgNPs.

**Figure 3:**
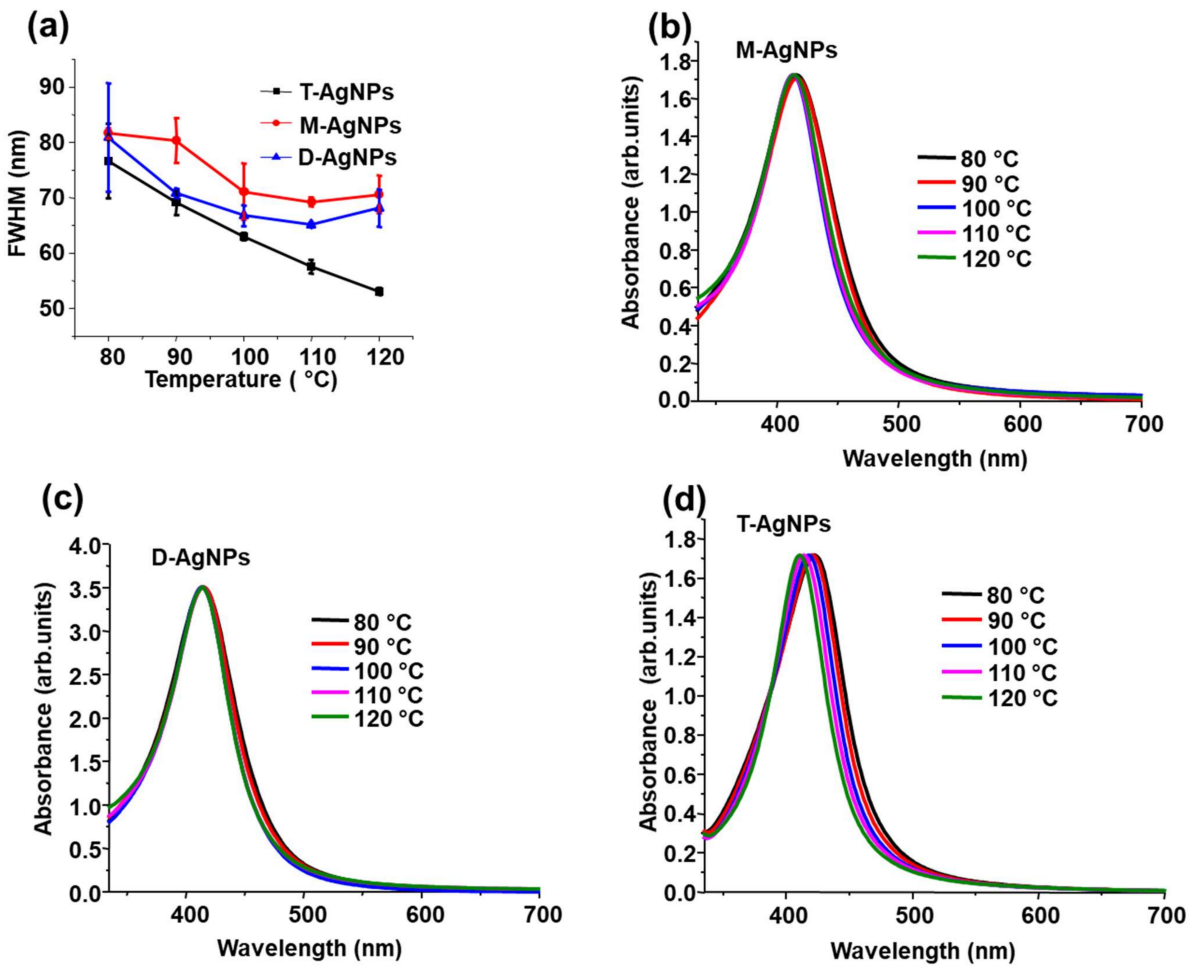
(**a**) FWHM (average of two sets of values from different batches of particles) of M-AgNPs, D-AgNPs, and T-AgNPs. UV-Vis spectra of: (**b**) M-AgNPs; (**c**) D-AgNPs; (**d**) T-AgNPs.

Figure 3a shows changes in the absorption maxima and changes in the FWHM of M-AgNPs, D-AgNPs, and T-AgNPs prepared at different temperatures. The surface plasmon bands of M-AgNPs, D-AgNPs, and T-AgNPs at different temperatures are shown in Figure 3b–d, respectively. The FWHM of the M-AgNPs and D-AgNPs decreases with temperatures from 80 °C to 100 °C and increases above 100 °C (Figure 3a–c). The FWHM of the T-AgNPs is narrower than that of the M-AgNPs and D-AgNPs, as follows from Figure 3d. This observation suggests that the trivalent ligands effectively stabilize the AgNPs and direct the isotropic growth of AgNPs in the presence of trivalent ligands more than for mono- and divalent ligands. This assumption is further supported by the maximum peak values deduced from absorption maxima (Figure 3 and Table 1). In the case of the T-AgNPs, the maximum gradually shifts from 423 nm to 411 nm when the temperature increases from 80 °C to 120 °C. In contrast, the FWHM of the M-AgNPs and D-AgNPs shifts only slightly from 417 to 415 nm and from 415 to 414 nm, respectively. This result is consistent with those reported in the literature, i.e., that the absorption of AgNPs depends on the size and shape of AgNPs [72,73]. Spherically and non-spherically shaped AgNPs show absorption bands at shorter and longer wavelengths, respectively [5,74]. The maxima of T-AgNPs, D-AgNPs, and M-AgNPs were observed at low wavelengths (below 450 nm), suggesting the occurrence of spherically shaped particles. The spectral shift of the maximum in T-AgNPs showed results consistent with those observed by Peng et al., [73], i.e., a blue-shift of the absorption maximum when AgNPs particles decreased in size from ~20 nm to ~12 nm. Further, the shift in absorption was supported by the multilayer Mie theory [73]. It has been previously reported that the width of the plasmon peak increased linearly with reciprocal particle size [72]. In the present study, we observed that the mono-, di-, and trivalent amine ligands changed the FWHM and peak maxima of AgNPs. By comparing the results obtained from the UV-Vis absorption measurements with previously reported results [5,72,74], it is clear that AgNPs are smaller in size and spherically shaped. To further confirm the spherical shape of AgNP, TEM measurements were carried out. 

**Figure 4:**
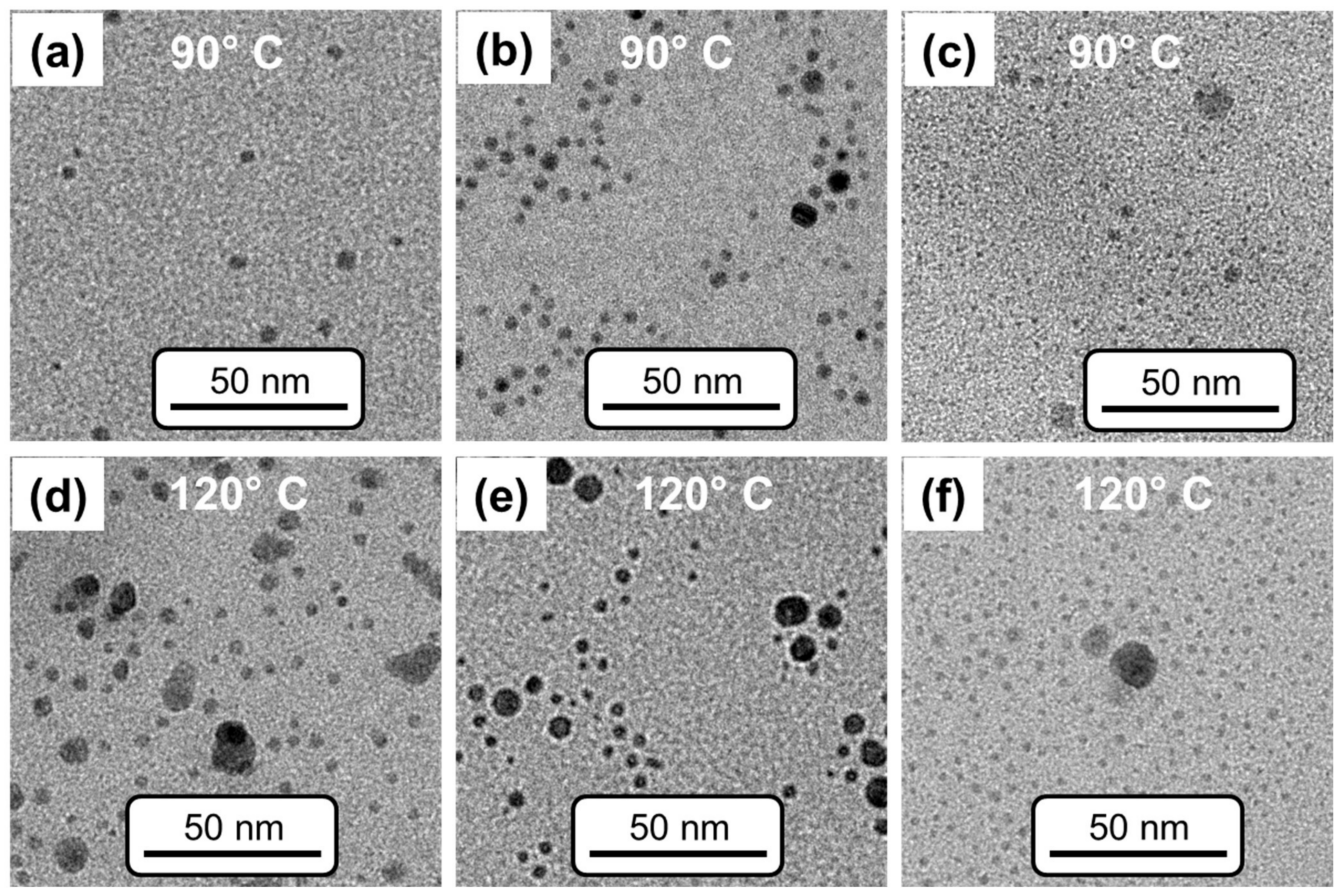
TEM images of M-AgNPs, D-AgNPs, and T-AgNPs, prepared at two different temperatures (90 °C (**a**–**c**) and 120 °C (**d**–**f**)), respectively.

The TEM images of M-AgNPs, D-AgNPs, and T-AgNPs prepared at 90 °C and at 200 °C are displayed in Figure 4a–c and Figure 4d–f, respectively. The size distributions from Figure 4 are shown in Figure S1 in the Supplementary Materials. The TEM images indicate varied sizes of AgNPs, from 1 to 25 nm in diameter. The diameters of the M-AgNPs vary between 1 and 12 nm (Figure 4 and Figure S1) at 90 °C (Figure 4a and Figure S1a) and 200 °C (Figure 4d and Figure S1d). A few ~25 nm particles are observed, which is evident from Figure S1a. The D-AgNPs show particle sizes in a range from 1 nm to 13 nm in diameter at 90 °C and 120 °C (Figure 4b,e and Figure S1b,e). At 120 °C, a bimodal distribution is found for D-AgNPs with particle fractions of less than and greater than 5 nm (Figure S1e). Particle sizes between 1 nm and 7 nm in diameter are observed for T-AgNPs at 90 °C and 120 °C, which follows from Figure 4c,f and Figure S1c,f, respectively. The TEM results reveal that T-AgNPs are more monodisperse than M-AgNPs and D-AgNPs, which is consistent with the narrower FWHM of UV-Vis absorption spectroscopy results. In addition, the TEM images confirm the spherical shape of AgNPs. This supports our assumption on the spherical shape of AgNPs as derived from the absorption maxima below 450 nm. 

Further, the observed narrowing effect of the AgNPs with differences in ligands, which is related to a decrease in FWHM with increasing reaction temperature, can be explained by a digestive ripening process [75,76]. The possible mechanism during the formation of AgNPs in the presence of mono- and multivalent ligands is proposed as follows, by considering the concept of a digestive ripening process: On reflux of the reactants, AgNPs with a wide size distribution form initially by consuming the metal salts. Solid materials (insoluble reactants) were observed at a lower temperature (80 °C) and dissolved with an increase in temperature. Thus, the initially formed polydispersed smaller and larger AgNPs might undergo a size modification process. The larger AgNPs are cut out into small AgNPs with ligands that are present on the surface of AgNPs. The stabilization of smaller AgNPs with ligands prevents the aggregation of AgNPs, which finally creates optimum-sized AgNPs. Thus, the digestive ripening process results in less polydisperse AgNPs. The narrow size distribution of T-AgNPs reveals that the digestive effect is evidently more effective in T-AgNPs than M-AgNPs and D-AgNPs. The formation of smaller T-AgNPs with lower polydispersity is more efficient than the D-AgNPs and M-AgNPs, this accounts for the sterically hindered trivalent ligands as compared with the less hindered divalent and monovalent ligands on AgNPs. 

### 3.2. Effects of Mono- and Multivalent Amine Ligands on the Formation of PtNPs and AgNPs by a One-Step Process

To investigate the effect of ligands on the formation of AgNPs and PtNPs at one specific temperature, mono- and multivalent ligands stabilized PtNPs and AgNPs were prepared via thermal reduction of Pt(acac)_2_ or Ag(acac) with HD at 200 °C and 120 °C in OE, respectively. These temperatures correspond to the final reaction temperature in the stepwise formation process of AgNPs and PtNPs, ranging from 80 °C to 120 °C and from 160 °C to 200 °C, respectively. 

During the preparation of M-PtNPs, the reaction mixture immediately turned a blackish brown color at 200 °C. In the case of the D-PtNPs and T-PtNPs, the reaction mixture color changed to blackish brown after 5 min. This color change suggests the formation of PtNPs. Since black precipitates were immediately found for M-PtNPs, this reaction mixture was heated only for 5 min. 

**Figure 5:**
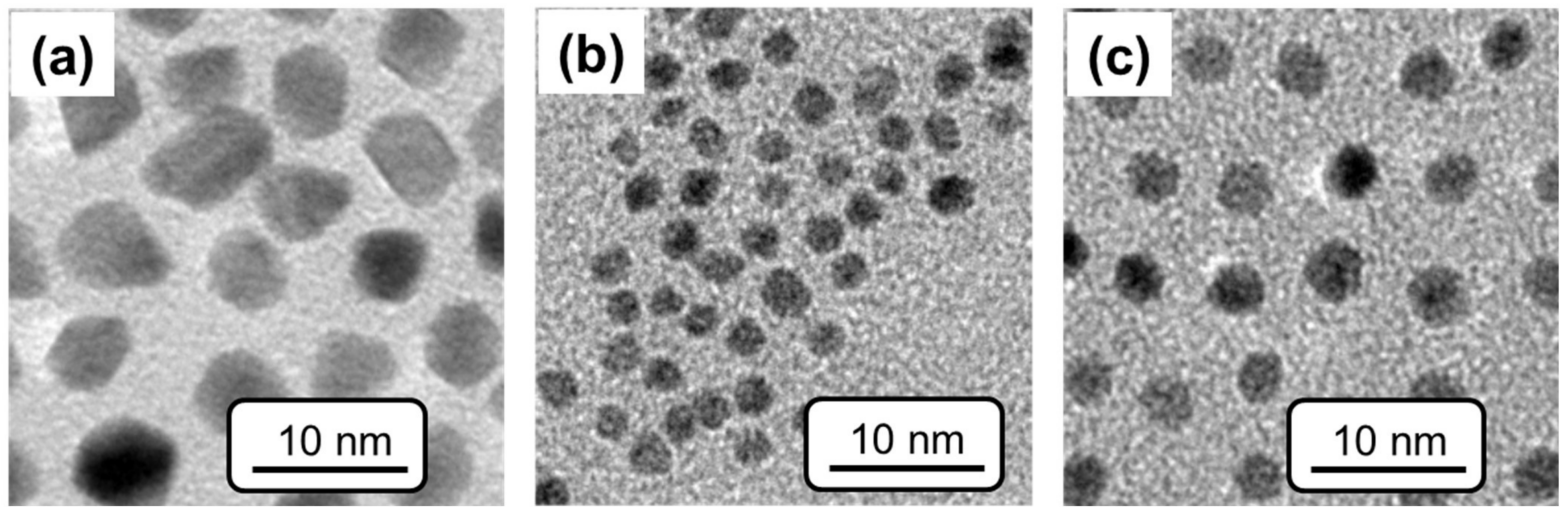
High magnification TEM images of: (**a**) M-PtNPs; (**b**) D-PtNPs; (**c**) T-PtNPs, synthesized at 200 °C.

The shapes and size distributions of the particles were investigated using the TEM measurements (Figure 5). The polyhedral shapes of the M-PtNPs are evident in Figure 5a, whereas the D-PtNPs (Figure 5b) and T-PtNPs (Figure 5c) are spherically shaped particles. The size distribution of each sample was obtained from the TEM images using the highest diameter of the M-PtNPs, and the spherical diameter of the D-PtNPs and T-PtNPs. The average size of the polyhedral structures of the M-PtNPs was determined as 5.7 ± 0.6 nm. The sizes of the spherically shaped D-PtNPs were determined as 2.6 ± 0.5 nm. The T-PtNPs were also spherically shaped with an average diameter of 3.6 ± 0.4 nm. The formation of polyhedral and spherically shaped PtNPs has been reproduced for the size distributions within ±0.05 nm. The quick color change of the reaction mixture during the synthesis of M-PtNPs suggests rapid growth of PtNPs in the presence of monovalent ligands. The spherically shaped D-PtNPs and T-PtNPs favor a slow and isotropic growth of PtNPs in the presence of divalent and trivalent ligands, respectively. The formation of black precipitates during the synthesis of M-PtNPs indicated the partial aggregation of PtNPs in the presence of monovalent ligands. However, these black precipitates were not observed during the synthesis of D-PtNPs and T-PtNPs. 

The formation of aggregates can be explained by considering differences in the affinity of the ligands used to the metal particles. Monovalent ligands have lower binding affinity than the di- or trivalent ligands [70,77,78,79,80]. Because of the low binding affinity of the monovalent ligands, they fail to provide steric stabilization of the PtNPs. Thus, partial aggregation was observed during the preparation of M-PtNPs. These aggregates were removed during the TEM sample preparation by filtering the dispersed M-PtNPs in CH_2_Cl_2_ via a PTFE (polytetrafluoroethylene) syringe filter (0.2 µm). Since no precipitation was observed for D-PtNPs and T-PtNPs, the corresponding TEM samples were prepared from CH_2_Cl_2_ dispersions without any filtration. The D-PtNPs and T-PtNPs were stable for at least one month and could be readily redispersed in CH_2_Cl_2_ without any notice of precipitation. 

It is well known from the literature that the nanoparticle shapes can be efficiently controlled by varying the ratios of ligand to metal precursor [50], the ligands [81,82], and the metal salts [83,84]. In this respect, the morphologies of PtNPs can be controlled by stabilizing agents. Here, the observed shape differences can be explained by the consideration of the ligand’s affinity. Due to the high affinity and avidity of multivalent ligands, they provide steric stabilization and direct the formation of thermodynamically stable spherically shaped PtNPs. Due to the low affinity of monovalent ligands, the particles are directed towards the kinetically controlled growth of polyhedral shapes. 

By performing the one-step process, AgNPs were prepared at 120 °C in the presence of mono- and multivalent ligands. The prepared M-AgNPs, D-AgNPs, and T-AgNPs were characterized by performing UV-Vis absorption spectroscopy.

**Figure 6:**
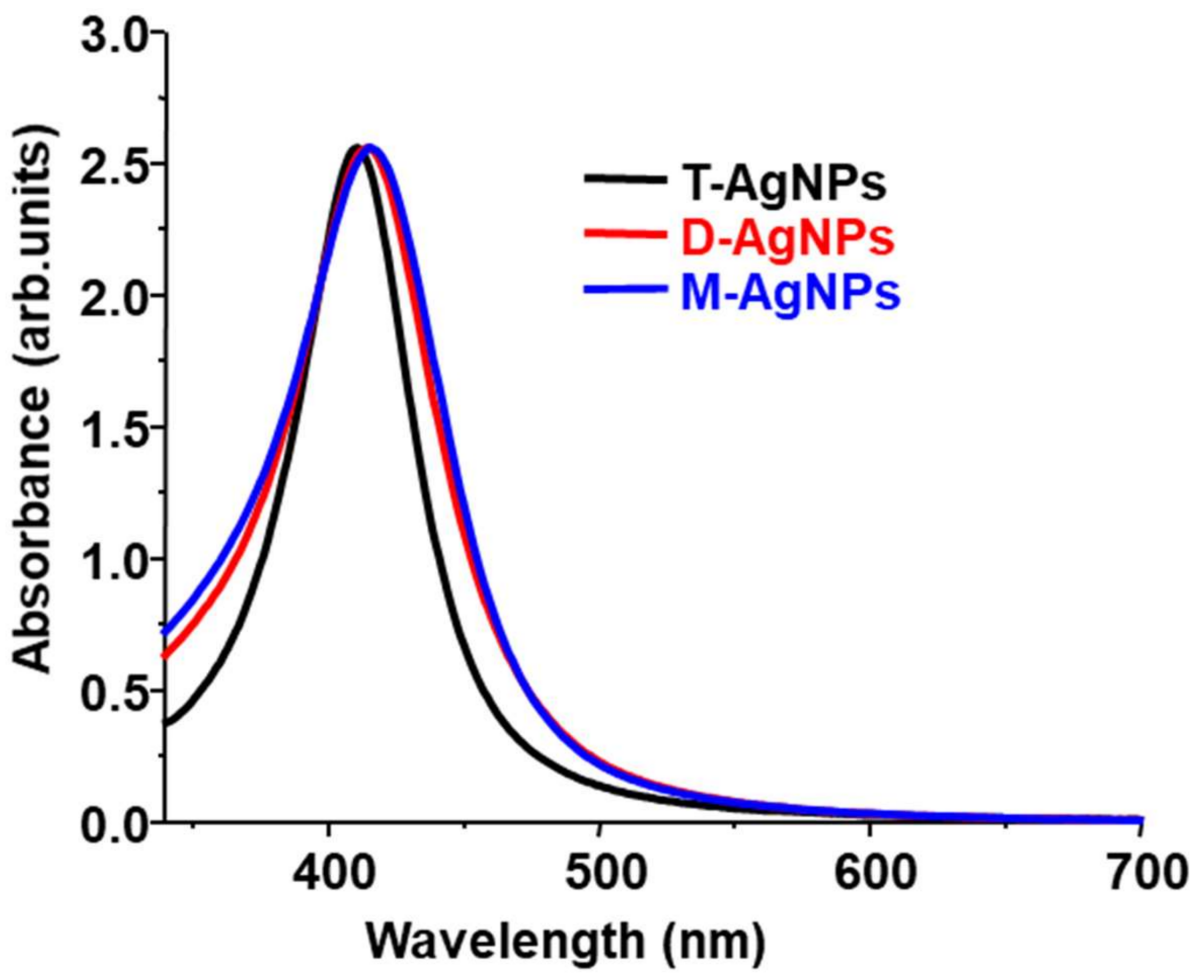
UV-Vis absorption spectra of T-AgNPs, D-AgNPs, and M-AgNPs prepared at 120 °C.

Figure 6 shows, in greater detail, the UV-Vis absorption spectra of the M-AgNPs, D-AgNPs, and T-AgNPs prepared at 120 °C. The FWHM of M-AgNPs and D-AgNPs was determined as ~70 nm, but in the case of the T-AgNPs, the value was found to be only ~50 nm, which was narrower as compared with those of M-AgNPs and D-AgNPs. The maximum of the localized surface plasmon resonance spectrum of the T-AgNPs was measured as 410 nm, whereas M-AgNPs and D-AgNPs yield maxima at 415 nm. The narrower FWHM of T-AgNPs indicates that T-AgNPs have a significantly narrower size distribution, and the lower absorption maximum suggests a slightly smaller average particle size. Since, the observed absorption peaks of T-AgNPs, D-AgNPs, and M-AgNPs were below 450 nm, the formation of spherically shaped AgNPs is suggested [75,76]. This is consistent with the results obtained for T-AgNPs, D-AgNPs, and M-AgNPs prepared by performing a stepwise process.

A unique approach is presented here for the preparation of amine ligand stabilized AgNPs and PtNPs via a one-pot synthesis process. To the best of our knowledge, the detailed mechanism for reducing metals at high temperatures into nanoparticles is still unclear. However, we assume that HD mediates the reaction, by releasing a molecule of water to form an aldehyde, and then the aldehyde reduces the metal salt [85]. The ligand and temperature effects on the formation of PtNPs and AgNPs were systematically investigated by performing the one-step and stepwise processes.

## 4. Conclusions

In this study, we report on a facile, stable one-pot synthesis of mono- and multivalent amine ligand-capped PtNPs and AgNPs. Following a stepwise process, temperature effects in the range from 160 °C to 200 °C on the formation of PtNPs in the presence of mono- and multivalent amine ligands were systematically investigated using TEM measurements. Our results revealed that M-PtNPs, D-PtNPs, and T-PtNPs at different temperatures revealed spherical particles regardless of the temperature and ligand multivalency. In contrast, during the one-step process at 200 °C, the M-PtNPs are polyhedrally shaped particles, while D-PtNPs and T-PtNPs are spherically shaped particles. The high binding affinity of the multivalent ligands facilitates the uniform growth of PtNPs and results in the formation of particles with a spherical shape. The investigations of temperature effects and ligand effects of AgNPs were studied by performing TEM and UV-Vis absorption spectroscopy. The formation of AgNPs with mono- and multivalent ligands by performing the stepwise process at temperatures ranging between 80 °C and 120 °C showed spherically shaped particles. However, the size distributions were narrowed by an increase in temperature and with ligand multivalency, and this was especially the case for AgNPs with trivalent ligands. This effect was explained using digestive ripening concepts, where the larger particles dissolve at elevated temperatures into smaller particles. By performing a one-step process at 120 °C, a narrower particle size distribution was obtained for T-AgNPs as compared with those for D-AgNPs and M-AgNPs. Thus, this work shows a versatile method to prepare AgNPs and PtNPs in a controlled manner. The obtained particles can be applied for applications such as electrocatalysis, catalysis, and other applications.

## Figures and Tables

**Table 1:** Maxima of the surface plasmon resonance spectra of M-AgNPs, D-AgNPs, and T-AgNPs prepared at 80 °C, 90 °C, 100 °C, 110 °C, and 120 °C.

Temperature (°C)	Maximum (nm)		
	M-AgNPs	D-AgNPs	T-AgNPs
80	417 ± 1	415 ± 1	423 ± 1
90	417 ± 1	415 ± 1	421 ± 1
100	416 ± 1	414 ± 1	418 ± 1
110	415 ± 1	414 ± 1	414 ± 1
120	415 ± 1	414 ± 1	411 ± 1

## Data Availability

Not applicable.

## Supplementary Materials

The following supporting information can be downloaded at: https://www.mdpi.com/article/10.3390/nano12132294/s1, The NMR details of divalent amine and trivalent amine ligands. Figure S1: Histogram of size distribution of silver nanoparticles with monovalent amine, divalent amine, and trivalent amine ligands that were prepared at 90 °C and 120 °C.
