# Supplementary material for: Controlled Synthesis of Platinum and Silver Nanoparticles Using Multivalent Ligands
Source: Nanomaterials (Basel). 2022 Jul 4;12(13):2294. doi: 10.3390/nano12132294 (PMC9268602; doi:10.3390/nano12132294)
Supplement: Supplementary file 1 [file nanomaterials-12-02294-s001.zip › nanomaterials-1783288-supplementary.pdf]

nanomaterials

Article

Controlled Synthesis of Platinum and Silver Nanoparticles Using Multivalent Ligands

Suguna Perumal [1,2,†], Raji Atchudan [3,†], Eckart Rühl [1,*] and Christina Graf [1,4,*]

1 Physikalische Chemie, Institut für Chemie und Biochemie, Freie Universität Berlin, 14195 Berlin, Germany; suguna.perumal@gmail.com
2 Department of Chemistry, Sejong University, Seoul 143-747, Korea
3 School of Chemical Engineering, Yeungnam University, Gyeongsan 38541, Korea; atchudanr@yu.ac.kr
4 Department of Chemistry and Biotechnology, Darmstadt University of Applied Sciences, 64295 Darmstadt, Germany
* Correspondence: ruehl@zedat.fu-berlin.de (E.R.); christina.graf@h-da.de (C.G.)
† These authors contributed equally to this work.

Characterization of flexible amine-functionalized multivalent ligands

Divalent Amine:

NMR-^{1}H (400 MHz, $CDCl_3$) δ: 0.88 (t, 3H, J = 6.7 Hz), 1.22 (s, 4H), 1.29 (s, 14H), 1.37-1.44 (m, 1H), 2.66-2.75 (m, 4H).

NMR-^{13}C (100 MHz, $CDCl_3$) δ: 14.0, 22.5, 26.9, 29.2, 29.5, 29.7, 29.9, 31.8, 43.6 (2C), 43.7.

Trivalent Amine:

NMR-^{1}H (400 MHz, $CDCl_3$) δ: 0.88 (t, 3H, J = 7.0 Hz), 1.20 (s, brs, 10H), 1.32 (brs, 10H), 2.58 (s, 6H).

NMR-^{13}C (100 MHz, $CDCl_3$) δ: 14.0, 22.5, 22.8, 29.2, 29.5, 30.6, 31.2, 31.7, 41.6, 44.7 (3C).

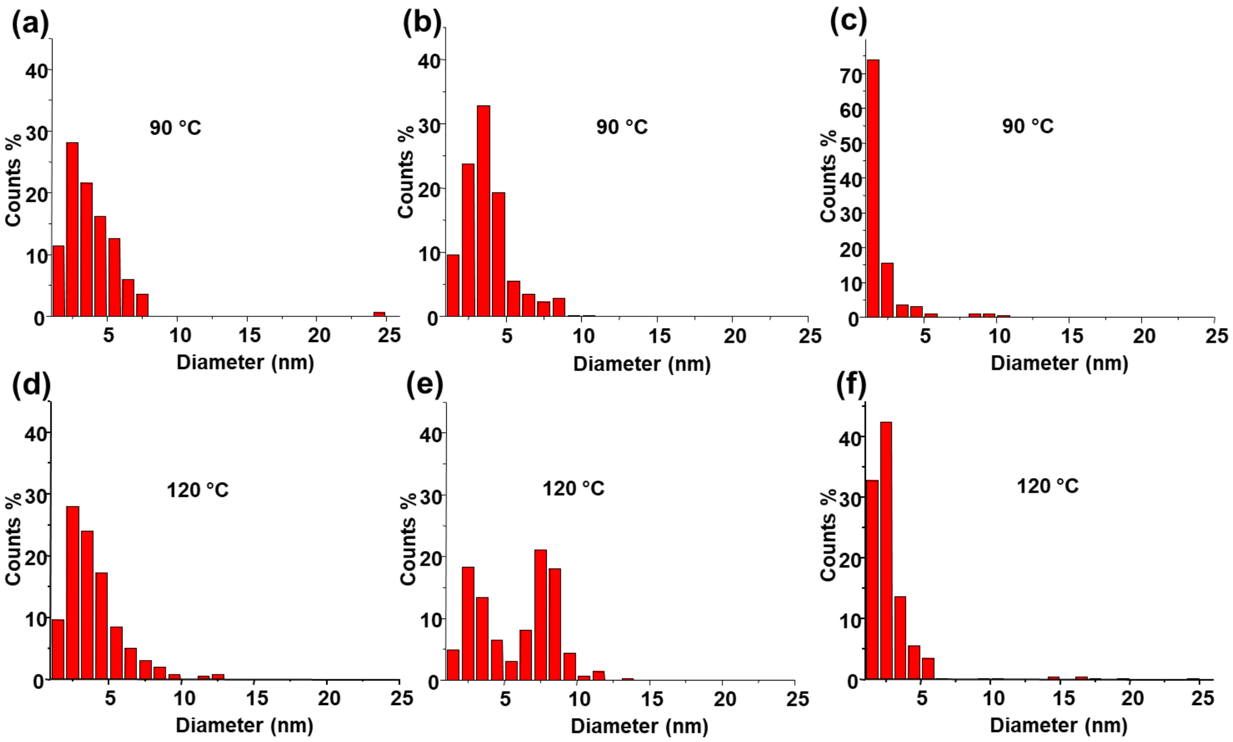

Figure S1. Histograms depict the **s**ize distribution of silver nanoparticles with monovalent ligands (M-AgNPs), silver nanoparticles with divalent ligands (D-AgNPs), and silver nanoparticles with trivalent ligands (T-AgNPs) at two different temperatures (90 °C (a-c) and 120 °C (d-f)), respectively.
